# Genetic Predisposition in NAFLD and NASH: Impact on Severity of Liver Disease and Response to Treatment

**DOI:** 10.2174/13816128113199990381

**Published:** 2013-09

**Authors:** Paola Dongiovanni, Quentin M Anstee, Luca Valenti

**Affiliations:** 1 Department of Pathophysiology and Transplantation, section Internal Medicine, Università degli Studi Milano, UO Medicina Interna 1B, Fondazione IRCCS Ca’ Granda Ospedale Maggiore Policlinico, Milan, Italy;; 2 Institute of Cellular Medicine, Newcastle University, Newcastle upon Tyne, United Kingdom.

**Keywords:** Non-alcoholic fatty liver disease, non-alcoholic steatohepatitis, polymorphism, genetic predisposition.

## Abstract

Liver fat deposition related to systemic insulin resistance defines non-alcoholic fatty liver disease (NAFLD) which, when associated with oxidative hepatocellular damage, inflammation, and activation of fibrogenesis, i.e. non-alcoholic steatohepatitis (NASH), can progress towards cirrhosis and hepatocellular carcinoma. Due to the epidemic of obesity, NAFLD is now the most frequent liver disease and the leading cause of altered liver enzymes in Western countries. Epidemiological, familial, and twin studies provide evidence for an element of heritability of NAFLD. Genetic modifiers of disease severity and progression have been identified through genome-wide association studies. These include the *Patatin-like phosholipase domain-containing 3* (*PNPLA3*) gene variant I148M as a major determinant of inter-individual and ethnicity-related differences in hepatic fat content independent of insulin resistance and serum lipid concentration. Association studies confirm that the I148M polymorphism is also a strong modifier of NASH and progressive hepatic injury. Furthermore, a few large multicentre case-control studies have demonstrated a role for genetic variants implicated in insulin signalling, oxidative stress, and fibrogenesis in the progression of NAFLD towards fibrosing NASH, and confirm that hepatocellular fat accumulation and insulin resistance are key operative mechanisms closely involved in the progression of liver damage. It is now important to explore the molecular mechanisms underlying these associations between gene variants and progressive liver disease, and to evaluate their impact on the response to available therapies. It is hoped that this knowledge will offer further insights into pathogenesis, suggest novel therapeutic targets, and could help guide physicians towards individualised therapy that improves clinical outcome.

## 1 INTRODUCTION

Liver fat deposition related to systemic insulin resistance (IR) defines non-alcoholic fatty liver disease (NAFLD) [1]. In susceptible individuals this maybe associated with oxidative hepatocellular damage, inflammation, and activation of fibrogenesis, i.e. non-alcoholic steatohepatitis (NASH) [2], potentially progressing towards cirrhosis and hepatocellular carcinoma [3]. Due to the epidemic of obesity and the metabolic syndrome, NAFLD is now the most frequent liver disease (prevalence 20-34%) and the leading cause of altered liver enzymes in Western countries [4, 5]. Although NASH is still an emerging health problem, it is already projected to become the leading cause of end-stage liver disease, liver transplantation and hepatocellular carcinoma within the next 10-20 years.

Epidemiological, familial, and twin studies provide evidence for an element of heritability of hepatic fat content, NAFLD, and *bona fide* metabolic cirrhosis [6, 7]. Recently, the inherited determinants of steatosis are beginning to be unrevealed using genome-wide association studies. These have identified *Patatin-like phosholipase domain-containing 3* (*PNPLA3*) gene variants as a major determinant of inter-individual and ethnicity-related differences in hepatic fat content independent of insulin resistance and serum lipids concentration [8].

Furthermore, a few large multicenter case-control studies demonstrate a role of genetic variants implicated in insulin signalling [9], oxidative stress [10, 11], and fibrogenesis [12] in the progression of NAFLD towards fibrosing NASH, and confirm that hepatocellular fat accumulation and insulin resistance are key operative mechanisms in the pathophysiology of NAFLD, and are closely involved in the progression of liver damage. 

The main purpose of this review is to provide an overview of what is known about the genetic predisposition to NAFLD and in particular to the progressive form of liver disease, NASH. In addition, we will outline the possible influence of genetic variation on the response to NASH treatment, an area where more studies are urgently needed. New genetic risk factors could prove useful for the clinical management of patients with NAFLD, as well as for other chronic liver diseases associated with steatosis, and for the identification of novel therapeutic targets for NASH, for which specific treatments are still lacking.

## 2 PATHOPHYSIOLOGY OF NAFLD AND NASH

A short overview of the pathophysiology of NAFLD and NASH is required to introduce the genetic determinants of disease pathogenesis and progression. The acronym NAFLD defines a wide spectrum of liver disease ranging from simple uncomplicated hepatic fat accumulation in the form of triglycerides exceeding 5% of liver mass (steatosis) in the absence of significant alcohol consumption to severe hepatitis characterized by steatosis, lobular inflammation, and hepatocellular damage and apoptosis with activation of fibrogenesis (steatohepatitis, NASH) [13], which can progress to cirrhosis and hepatocellular carcinoma [3]. Hepatic fat accumulation results from an unbalance between triglycerides acquisition and removal [14], and initially represents a protective mechanism to protect hepatocytes from the toxicity resulting from an increased flux of free fatty acids (FFAs) to the liver [15]. Several lines of evidence support the hypothesis that most of the FFAs accumulated as triglycerides during steatosis derive from increased peripheral lipolysis [16] related to adipose tissue insulin resistance [17], followed by increased lipogenesis induced by hyperinsulinemia and diet. Indeed, the major risk factor for NAFLD is systemic insulin resistance due to central obesity and the metabolic syndrome [1, 18]. Steatosis *per se* may then further contribute to hepatic insulin resistance, exacerbating these metabolic disturbances and increase the risk of other extra-hepatic complications including cardiovascular disease [19, 20]. Impaired ability to secrete lipoproteins [21] and changes in fatty-acid oxidation also contribute to hepatic fat accumulation. 

**Fig. (1):**
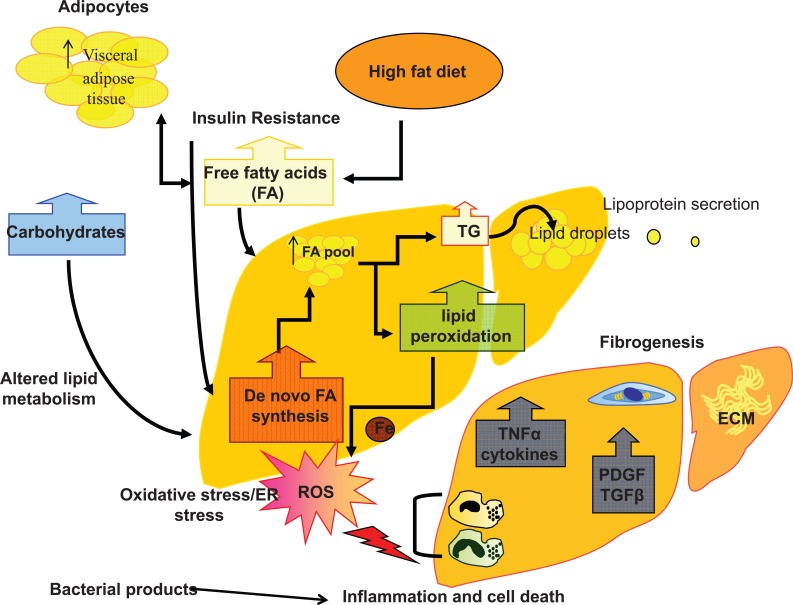
**Mechanisms involved in the pathogenesis of NASH**. NAFLD is characterized by the hepatic fat accumulation resulting from an unbalance between triglycerides acquisition and removal. Most of free fatty acids (FFAs) that are stored as triglycerides during hepatic steatosis derive from peripheral lipolysis related to adipose tissue insulin resistance, followed by *de novo* lipogenesis induced by hyperinsulinemia, and diet. In the liver, FFAs can be catabolized through β-oxidation, re-esterification to triglycerides and stored as lipid droplets, or exported as very low density lipoproteins (VLDL). Impaired ability to secrete lipoproteins and decreased β-oxidation due to mitochondrial damage (expecially in the presence of NASH) may play a role in hepatic fat accumulation. Long-term injury arising from i) hepatocellular triglycerides storage and lipotoxicity, ii) hepatocellular oxidative stress secondary to free radical produced during β- and omega- oxidation of FFAs, iii) inflammation triggered by endotoxin, iv) cytokines release, v) and endoplasmic reticulum (ER) stress lead in the end to inflammation, perpetuation of cellular damage, and activation of fibrogenesis.

**Fig. (2):**
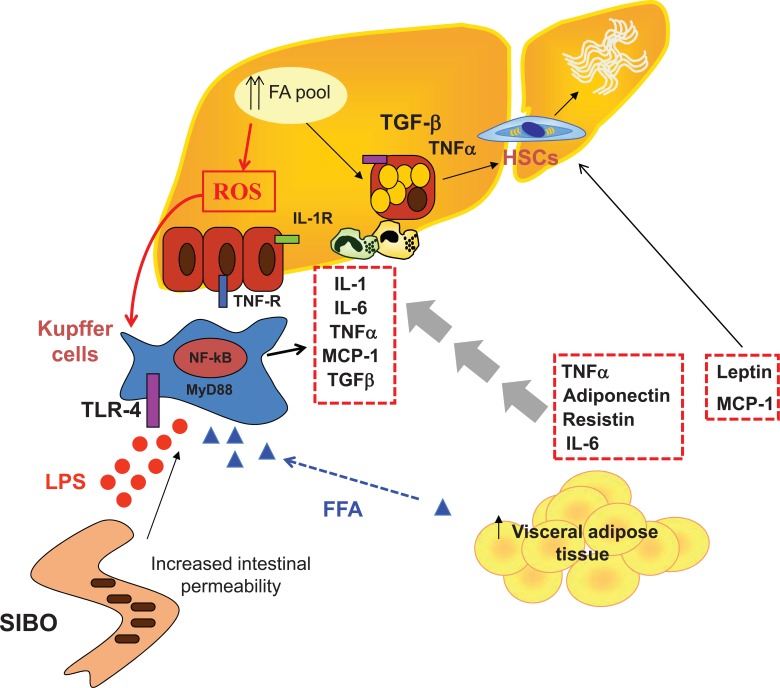
Inflammation in NAFLD. Obesity and NAFLD are directly associated with activation of inflammatory pathways. Hypertrophic adipocytes release chemokines and proinflammatory cytokines including TNFα, IL-6, resistin and MCP-1. Chemokines recruit macrophages, especially in visceral adipose tissue. Adipose tissue macrophages produce inflammatory cytokines such as TNFα, IL-6 and IL-1β. These inflammatory changes in adipose tissue induce adipocytokine dysregulation: a decrease in insulin sensitizing and anti-inflammatory adipocytokines as adiponectin, and an increase in pro-inflammatory cytokines such as TNFα, interleukins and resistin. Extracellular free fatty acids (FFAs) as well as bacterial endotoxins activate Kupffer cells by engaging the Toll-like receptor 4 (TLR4). Upon TLR ligation, MyD88, an adaptor molecule, is recruited to transmit the signals that activate NF-kB and JNK. Activated Kupffer cells produce inflammatory cytokines such as TNFα and IL-1β, chemokines such as MCP-1 and ROS leading to liver damage. Acute loss of hepatocytes triggers a compensatory proliferative response in surviving hepatocytes. However, in chronic fatty liver many hepatocytes have sustained oxidative damage that inhibits progression to the cycle and regeneration. Moreover fatty hepatocytes have reduced proliferative capacity. Damaged hepatocytes release several factors including ROS, cytokines, chemokines that recruit inflammatory cells into the liver. Once in the liver, these inflammatory cells release cytotoxic factors that increase hepatocytes death. Other hepatocytes-derived factors activate hepatic stellate cells (HSC), which produce more extracellular matrix (ECM) leading to matrix accumulation and fibrogenesis. HSCs also release fibrogenic cytokines with autocrine and paracrine effects, including TGF-β1, and over-express tissue inhibitors of metalloproteinase, which promote ECM accumulation by inhibiting matrix degradation.

Development of NASH has been classically explained by the occurrence of a so-called “second-hit”, leading to the activation of inflammation, in the context of hepatic steatosis [22]. This second insult is likely to actually represent a combination of insults related to direct hepatic lipotoxicity, hepatocellular oxidative stress secondary to free radicals produced during β- and ω- oxidation of FFAs, inflammation triggered by endotoxin engaging TLR-4 receptors in Kupffer cells and hepatocytes due to increased intestinal permeability, bacterial overgrowth and altered intestinal flora [23-25], cytokines release, and endoplasmic reticulum stress. These combine to produce inflammation, cellular damage, and activation of fibrogenesis [26]. A working model of NASH pathogenesis is presented in (Fig. **1**), whereas the role of inflammation is presented in greater details in (Fig. **2**).

## 3 EVIDENCE OF HERITABILITY OF NAFLD AND NASH

A possible explanation for the observed inter-individual variability in the susceptibility to NAFLD and progressive NASH is provided by heritability [26]. In addition to evidence of heritability provided by epidemiological, familial, and twin studies [6, 7, 27-29], clinical case series have also shown familial clustering of NAFLD [29]. Although shared environmental risk factors may contribute to development of steatosis, the variation in NAFLD phenotypic expression in persons with similar risk factors implicates a genetic contribution. Recently, a familial aggregation study of fatty liver in overweight children with and without NAFLD showed that liver fat fraction and in particular the condition of fatty liver are strongly heritable traits [6]. In addition, a family study of 157 individuals with familial combined hyperlipidemia (FCHL) showed that ALT levels and the prevalence of fatty liver were increased not only in FCHL probands but also in their relatives, suggesting the presence of a genetic component [30].

**Fig. (3):**
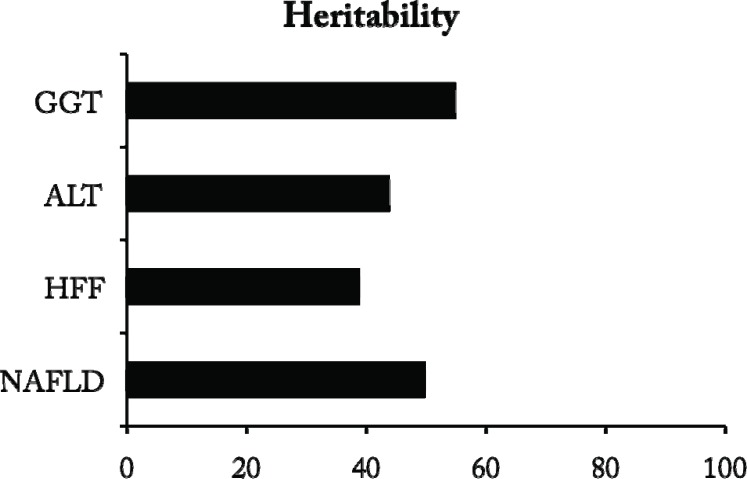
Heritable components of non-alcoholic fatty liver disease (NAFLD) and liver indices associated with steatosis. Average values of studies reported in the manuscript are presented. HFF: hepatic fat fraction; ALT: alanine transaminase; GGT: gamma-glutamyl transferase.

Twin studies confirmed that, in subjects without evidence of alcohol abuse or viral hepatitis, alanine transaminases (ALT) levels, mostly reflecting liver fat content, were a heritable trait, with genetic factors explaining almost up to 60% of variability [27]. A study population among Danish twins identified substantial heritability (35-61%) for levels of biochemical liver indices including ALT, gamma-glutamyl peptidase (GGT), the remaining variation being attributed to environmental factors [31]. Furthermore, GGT levels have also been shown to represent a highly heritable trait (roughly 50%), which had a significant covariance with risk factors for NAFLD determining the metabolic syndrome, such as IR, lipid levels, and diastolic blood pressure [32], and several studies demonstrated a role of genetic factors in the pathogenesis of metabolic alterations typical of hepatic IR and NAFLD [33], although some genetic risk factors for NAFLD are specific for hepatic fat content (see below). The estimates for heritable components of liver fat and liver enzymes associated with steatosis are presented in (Fig. **3**). However, it should be underlined that a recent study concluded that ultranosonographically detected NAFLD has a low heritability in Hungarian twins [34].

In line with a strong role of genetics in the pathogenesis of NASH, racial and ethnic differences have been reported in the prevalence of NAFLD, NASH and cryptogenic cirrhosis, which is believed to represent an evolution of NASH in the majority of cases [4, 35]. In the US for example, Hispanic subjects are at higher risk than subjects of European descent, whereas African-Americans are protected independently of diabetes and BMI [7]. The results of the Insulin Resistance Atherosclerosis Study (IRAS) Family Study with 1,142 participants of Hispanic or African-American descent was also consistent with a role of heritability in the pathogenesis of NAFLD [36]. 

## 4 *PNPLA3* I148M IS A MAJOR RISK FACTOR FOR NAFLD AND NASH

A major determinant of the inter-individual and ethnicity-related differences hepatic fat content quantified by magnetic resonance spectroscopy was identified by a genome-wide association scan (GWAS) of non-synonymous sequence variation reported in 2009, the rs738409 C>G SNP in the *Patatin-like phospholipase domain-containing 3 (PNPLA*3) gene, encoding for the isoleucine to methionine variant at protein position 148 (I148M) [8]. PNPLA3, also called adiponutrin, encodes a 481 amino acid protein expressed in the endoplasmic reticulum and at the surface of lipid droplets in hepatocytes and adipocytes, which is induced in the liver after feeding and during insulin resistance by fatty acids and the master regulator of lipogenesis SREBP-1c [37]. Although the mechanism and the physiological substrates remain an area of active research, the common I148M variant appears to disrupt the phospholipase activity of the enzyme, thus likely altering lipid catabolism, but it might also acquire new functions [38], and it has recently been reported to increase the synthesis of phospholipids [39]. Importantly, the association between PNPLA3 genotype and steatosis is independent of insulin resistance and serum lipids concentration [8], but appears to modify response to nutritional and lifestyle factors including obesity.

The association of the G allele encoding for the protein 148M variant with hepatic fat content has been confirmed in several studies [8, 40-49], and by a recent meta-analysis [50]. Sookoian *et al* reported an association between the 148M allele and increased severity of NAFLD [44] and we demonstrated that the frequency of 148M variant was significantly higher in NAFLD patients compared to healthy controls with a near 3.3-fold increased risk in Italian and UK subjects carrying the GG genotype [51]. The rs738409 SNP influenced both the presence of NASH and the severity of fibrosis in NAFLD patients with histological evaluation of liver damage, independent of body mass, diabetes and the previously demonstrated effect on NASH. The association of the I148M variant with progressive liver disease was independent of the predisposition to increased steatosis, thus suggesting that it influences the regulation of proinflammatory lipid mediators [52-54]. The effect of the rs738409 variant on liver fat and liver enzymes was apparent early in life [55, 56] and synergized with other risk factors of NAFLD. In a large cohort of Italian obese children with histologically proven NAFLD, the rs738409 G allele was the strongest determinant of steatosis severity, and in patients with the GG genotype severe steatosis was associated with increased lobular inflammation, hepatocellular ballooning and NASH [57], suggesting that the rs738409 genotype may represent a critical factor that determines whether the increased free fatty acids flux related to obesity translates into mild steatosis or progressive NASH in obese children. 

The coexistence of the rs738409 G risk allele (148M) and an independent environmental stressor such as obesity [58] or chronic alcohol consumption [59], is associated with elevated serum alanine transaminase levels and higher liver damage, suggesting that these stressors appear to uncover the association between 148M and hepatic injury. Indeed, the magnitude of the association between the I148M *PNPLA3* variant and liver enzymes was related to abdominal fat mass [60, 61], and to high dietary carbohydrate and sugar consumption [62]. The rs738409 *PNPLA3* genotype also influences steatosis development in chronic hepatitis C patients and is independently associated with cirrhosis and other steatosis-related clinical outcomes, such as lack of response to antiviral treatment and possibly hepatocarcinoma [63-66], and with of cirrhosis and hepatocellular carcinoma in patients with alcohol abuse [59, 67-71]. Importantly, it has recently been reported that the I148M *PNPLA3* variant is a risk factor for the development of hepatocellular carcinoma in severely obese subjects from Northern Europe [72].

**Fig. (4):**
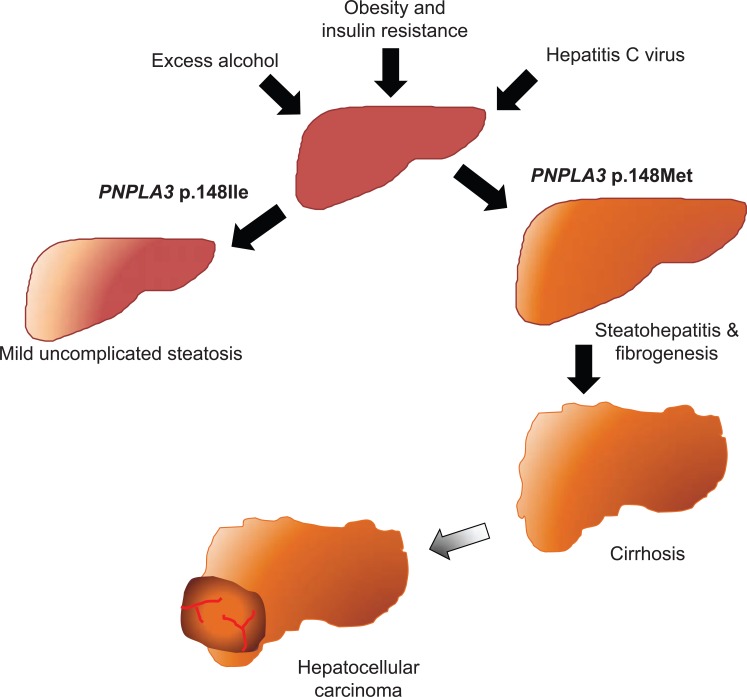
The I148M PNPLA3 mutation and progressive liver disease. A simplified working model showing the influence of the I148M PNPLA3 variant on the progression of chronic liver disease.

Therefore, current evidence suggests that the *PNPLA3* I148M variant is a genetic determinant of liver damage progression associated with steatohepatitis, which may be triggered by a number of factors including obesity, IR, excessive alcohol intake, and chronic hepatitis C (Fig. **4**) [53].

## 5 OTHER GENETIC VARIANTS INFLUENCING NAFLD SUSCEPTIBILITY IDENTIFIED BY GENOMEWIDE SCANS

Besides confirming that rs738409 of PNPLA3 is the major common genetic risk factor of NAFLD, a recent meta-analysis of combined GWAS datasets identified four other SNPs associated with liver fat content and other aspects of the NAFLD phenotype. These were localized in or near the genes neurocan (NCAN, SNP rs2228603), protein phosphatase 1, regulatory (inhibitor) subunit 3B (PPP1R3B, SNP rs4240624), glucokinase regulator (GCKR, SNP rs780094) and lysophospholipase-like 1 (LYPLAL1, SNP rs12137855). NCAN is involved in the regulation of cell adhesion and likely lipoprotein metabolism, and was also associated with histologically validated steatosis in a replication study. GCKR, a regulator of glucose metabolism and LYPLAL1, which exerts a complementary function to the PNPLA3 protein in triglyceride breakdown, were also associated with histologically assessed lobular inflammation and/or fibrosis [73]. The rs780094 GCKR polymorphism is in strong linkage disequilibrium with rs1260326, encoding for the P446L protein variant, which influences the ability of GCKR to inhibit glucokinase in response to fructose-6-phosphate, thereby resulting in a constant increase in hepatic glucokinase activity and glucose uptake by the liver [74]. Unrestricted hepatic glycolysis associated with carriage of the minor 446L allele leads on one hand to lower glucose and insulin levels, but on the other hand to increased levels of malonyl-CoA, which in turn may favor hepatic fat accumulation by serving as a substrate for lipogenesis and by blocking fatty acid oxidation through the inhibition of carnitine-palmytoil transferase-1 (CPT-1). The combined effects of PNPLA3 I148M and GCKR P446L polymorphisms has been proposed to explain up to one third of variability in liver fat content amongst obese children of European descent [75, 76].

To date one other GWAS has been reported. This identified variants conferring a predisposition to disease progression in a small cohort of patients with histologically proven NAFLD [77]. The study highlighted an association between severity of histological NAFLD activity score and SNP rs2645424 on chromosome 8, in the gene encoding farnesyl diphosphate farnesyl transferase 1 (FDFT1), an enzyme involved in cholesterol biosynthesis. Other associations observed included rs343062 on chromosome 7 with degree of fibrosis, and rs1227756 on chromosome 10 in the COL13A1 gene, rs887304 on chromosome 12 in the EFCAB4B gene with lobular inflammation. It was however perhaps surprising that this study did not identify PNPLA3 given that it has been repeatedly validated in some many other studies. These findings therefore require validation. 

## 6 GENETIC FACTORS INFLUENCING LIVER DISEASE PROGRESSION IN NAFLD FROM CANDIDATE GENE STUDIES

### 6.1 Variants Involved in the Regulation of Lipid Metabolism 

The hallmark of hepatic steatosis is triglyceride (TG) accumulation within hepatocytes caused by alterations in hepatic lipid metabolism changing the balance between the pathways of uptake, synthesis, degradation and secretion on a background of systemic insulin resistance [78]. Genes that affect hepatic fat storage and mobilization are therefore likely candidates to influence the development and progression of NAFLD as are variants of transcription factors controlling lipid metabolism in the liver and adipose. Peroxisome proliferator-activated receptor-alpha (PPARα) is a member of the nuclear hormone receptor superfamily. A molecular target of long chain fatty acids, eicosanoids and fibrates [79], it is highly expressed in tissues that catabolize fatty acids such as the liver and skeletal muscle. Under condition of increased hepatic fatty acid influx or decreased fatty acid efflux, PPARα activation prevents the accumulation of triglycerides by increasing the rate of fatty acid catabolism. PPARα downregulation is involved in NASH pathogenesis by reducing FFA catabolism [80]. The Val227Ala SNP in the PPARα gene may be implicated in the pathogenesis of NAFLD and it could play a protective role against the development of obesity [81]. It has been hypothesized that the substitution of Valine to Alanine at codon 227 causes a functional change in PPARα and that the Ala227 isoform has higher activity than the Val227 isoform [82]. However, in Italian subjects the Leu162Val PPARα loss-of-function polymorphism did not influence the risk of NAFLD, where it was associated with IR but not histologically assessed disease severity [83], suggesting that the risk related to increased insulin resistance may be balanced by the protective effect of decreased oxidative stress. 

Peroxisome proliferator-activated receptor-gamma (PPARγ), the molecular target of glitazones, is highly expressed in adipose tissue and regulates adipocyte differentiation, FFA uptake and storage. Pharmacological activation of PPARγ improves insulin resistance in diabetes and has been reported to decrease liver damage in NAFLD by restoring adipose tissue insulin sensitivity, decreasing FFA flux to the liver [84, 85]. The Pro12Ala loss-of-function SNP in PPARγ2, which is thought to induce a modest impairment of transcriptional activation due to decreased DNA-binding affinity, was associated with a reduction of PPARγ activity in adipose tissue as well as decreased IR and diabetes in Caucasians [86]. The data in liver disease are conflicting however, Rey *et al* showed a significantly higher risk of developing histological necro-inflammation in alcoholic liver disease patients carrying the 12Ala allele but found that the 12Ala allele was not associated with the progression of liver disease in NAFLD patients [87]. Similarly, the 12Ala allele was not associated with NAFLD susceptibility, liver damage or IR in 212 Italian patients with NAFLD [83]. However an association with carriage of the minor PPARγ haplotypes encompassing the 12Ala allele was reported with increased risk of progressive liver disease in a US cohort of similar size [88].

Another interesting candidate is represented by Lipin1 (LPIN1), a phosphatidate phosphatase that is highly expressed in adipose tissue, is involved in the metabolism of phospholipids and triacylglycerol, and is required for adipogenesis and the normal metabolic flux between adipose tissue and liver, where it also acts as an inducible transcriptional co-activator to regulate fatty acid metabolism [89]. LPIN1 mRNA expression in the liver and adipose tissue has been positively associated with body mass and IR. LPIN1 SNPs and haplotypes that may confer variability in the protein activity have been associated with several components of the metabolic syndrome, including body mass, insulin levels, resting metabolic rate and to responsiveness to insulin sensitizers [90, 91]. Whereas in a case-control population study of 17538 Danes LPIN1 variants and haplotypes did not influence type 2 diabetes, obesity, or related quantitative metabolic phenotypes [92]. However, in a recent meta-analysis conducted in 8504 subjects the LPIN1 rs13412852 T allele was associated with lower BMI and insulin levels [93], confirming that it possibly represents a protective factor towards metabolic syndrome alterations.

We evaluated whether the LPIN1 rs13412852 C>T polymorphism was associated with NASH and fibrosis in pediatric Italian patients with NAFLD, finding that the TT genotype was under-represented in pediatric but not in adult patients with NAFLD, was associated with less severe dysplipidemia, and that children with this genotype had a trend for a lower prevalence of NASH and significantly less severe liver damage independently of PNPLA3 genotype and other risk factors [94]. Although independent validation of these results is required, these data suggest that LPIN1 genotype may predispose to progressive NASH at early age by influencing lipogenesis and lipid metabolism.

Hepatic uptake of fatty acids (FA), as one of the routes to development of steatosis, is of clinical relevance. Fatty acid transport proteins (FATP) hold a crucial role in mediating FA uptake in different tissues [95-97]. In the liver two different FATP isoforms are predominantly expressed; FATP2 and FATP5 [98]. The FATP5 gene encodes a multifunctional protein which increases the hepatic uptake of FA and activates very long-chain fatty-acids and has bile-CoA ligase activity [99, 100]. Overexpression as well as inhibition of FATP5 in cell culture and experimental animals underline its function in hepatic fatty acid trafficking [101, 102]. Furthermore, mice lacking FATP5 have defective bile acid conjugation and are protected from obesity [100]. FATP5 silencing reverses diet-induced NAFLD and improves hyperglycemia in mice [101]. 

Since variations in the promoter region may alter the transcriptional activity [103] we investigated the association of a FATP5 promoter polymorphism with parameters of the fasting and postprandial lipid and glucose metabolism in a cohort study and in subjects with histologically proven NAFLD. A total of 716 male subjects from the Metabolic Intervention Cohort Kiel (MICK) and 103 male subjects with histologically proved non-alcoholic fatty liver disease (NAFLD) were genotyped for the rs56225452 FATP5 polymorphism and phenotyped for features of the metabolic syndrome. In the MICK cohort, ALT levels, postprandial insulin levels and triglyceride concentrations were higher in subjects carrying the rare A-allele than in GG homozygotes. Accordingly, the insulin sensitivity index determined after a mixed meal and standardized glucose load was lower in A-allele carriers. NAFLD cases carrying allele A were presented with also higher ALT activities. In NAFLD subjects the association of BMI with the degree of steatosis and glucose concentration differed across FATP5 promoter polymorphisms [104]. Therefore, the FATP5 promoter polymorphism rs56225452 seems to be associated with higher ALT levels, insulin resistance and dyslipidemia in the general population. The impact of the BMI on the severity of steatosis in NAFLD cases seems to depend partly on the FATP5 polymorphism, but additional studies are needed to define the association with progressive liver damage. 

Synthesis of phosphatidylcholine is required for VLDL formation, when it is not available fat droplets accumulate in the cytosol of hepatocytes [105], [106]. This observation underpins the use of choline deficient diets as a well-recognised animal model of NASH [107, 108]. Thus, modifier genes of choline metabolism provide another source of candidates for study. Phosphatidylethanolamine N-Methyltransferase (PEMT) catalyzes the *de novo* synthesis of phosphatidylcholine in the liver [109]. Synthesis of new phosphatidylcholine molecules is required for VLDL formation and when they are not available fat droplets accumulate in the cytosol of hepatocytes [105], [106]. *PEMT* knockout mice do not display any PEMT activity in the liver and depend completely on dietary choline intake [110, 111], and when fed a choline-deficient diet develop severe steatosis [112]. Song *et al*. identified a non-synonymous SNP in the *PEMT* gene (523 G>A in exon 8), which results in a loss-of-function valine to methionine (V175M) substitution in the encoded protein. A higher occurrence of the low-activity 175M variant was found in 28 subjects of mixed ethnicity with biopsy confirmed NAFLD compared to 59 controls, suggesting that this SNP may confer susceptibility to NASH [113]. Dong *et al*. found that carriage of the Val175Met variant allele was significantly more frequent in 107 patients with biopsy-proven NASH than in 150 healthy volunteers and also found that non-obese carriers of the Val175Met variant were at increased risk of NASH [114]. In contrast, Jun *et al*. did not find any difference in *PEMT* genotype frequency between NAFLD patients (n=195) and controls (n=393) [115] and Romeo *et al* also failed to identify an association between the V175M allele and hepatic triglyceride content in the Dallas Heart Study (DHS) multiethnic cohort of 2349 individuals [116]. Together these findings suggest that it is unlikely that the Val175Met mutation represents a major contributor to NASH susceptibility, although the effect of altering genetic background may have been a contributory factor. 

A defect in lipid export as lipoprotein may also contribute to the pathogenesis of steatosis [21]. Microsomal triglyceride transfer protein (MTTP) is necessary for assembly and secretion of VLDL from hepatocytes [117]. It has a key role in lipoprotein assembly by transferring triglycerides, to nascent apolipoproteins B. Abetalipoproteinaemia, a rare autosomal recessive disease caused by mutations in the coding region of the MTTP gene, results in very low total cholesterol, undetectable plasma apoB levels and fat malabsorption, and is characterized by liver steatosis although this seldom progresses to steatohepatitis [118, 119]. A common functional SNP in the *MTTP* gene promoter (-493G/T) has been described [120], with the G allele associated with decreased MTTP transcription, less export of triglycerides from hepatocytes, and greater intracellular triglycerides accumulation. Namikawa *et al*. showed that NASH patients had a higher incidence of the G allele and of the G/G genotype compared to controls, even if the number of both patients and controls included in the study was limited [121]. Moreover, the stage of NASH was more advanced in Japanese patients with the G/G genotype than in patients with G/T genotype. A relatively small study from Italy demonstrated that the -493 G/T *MTTP* SNP influences liver disease and postprandial lipid metabolism in NASH. Patients with the G/G genotype were found to have more severe liver disease and a more atherogenic postprandial lipoprotein profile, in spite of similar degrees of adiposity and insulin resistance, adipokine profile and dietary habits [122]. In 40 non-diabetic normo-lipidemic NASH patients compared to 40 healthy controls, the -493 G/T polymorphism modulated beta-cell function, an effect mediated by postprandial HDL-C and oxLDL metabolism [123]. In 271 French patients with type II diabetes the -493 G/T SNP was associated with increased liver enzymes and increased susceptibility to steatohepatitis, however this study provided only indirect evidence of a link between *MTTP* genotype and NASH, as liver biopsy specimens were not available and the authors adopted raised ALT as a surrogate for NASH [124]. This potential association was not supported by a Brazilian study in 131 patients with biopsy proven disease compared to 141 healthy volunteers: the presence of at least one -493 G allele was only marginally different between NASH and simple steatosis [125] (Tables **1**, **2**, and **3**).

Variants in apolipoproteins influencing serum lipid metabolism might be involved as well. Apolipoprotein E (ApoE) is a plasma protein involved in lipid transport and metabolism [126]. Three alleles, ε2, ε3 and ε4, at the ApoE locus determine three isoforms, ApoE2, ApoE3 and ApoE4, resulting in six ApoE genotypes (E2/2, E3/3, E4/4, E2/3, E2/4, E3/4). These isoforms differ by single amino acid substitution at position 112 and 158 of ApoE, which lead to a different association with lipoproteins and affinity for the LDL receptor [126, 127]. Homozygosity for the ε2 allele is associated with hyperlipidemia, but no significant difference in ApoE genotypes and allele frequencies was observed between NAFLD patients and controls. Nevertheless, comparing non obese patients with controls it was found that the ε2 allele and the ε2/3 genotype were more prevalent in the control group, suggesting that occurrence of this allele and of this genotype may be protective against the development of NAFLD [128]. In line with these data, the ApoE3/3 genotype was associated with an increased risk of NASH in a cohort of Turkish patients, whereas the ApoE3/4 genotype had a protective effect [129]. Interestingly, Price *et al.* reported that in patients with persistent hepatitis C virus (HCV), which circulates in the plasma associated with VLDL, allowing the virus to enter target cells via lipoprotein receptors [130], the common genetic variations at the ApoE locus influence the outcome of HCV infection, with the ε2 and ε4 alleles favoring viral clearance [131]. 

Apolipoprotein C3 (ApoC3) is another major constituent of plasma very low density lipoprotein (VLDL), chylomicrons and HDL-C which inhibits lipoprotein lipase and triglyceride clearance [132]. Petersen *et al* reported that two common ApoC3 T-455C and C-482T promoter SNPs, which hamper the regulation of apolipoprotein C3 expression by insulin signalling via FOXO1 phosphorylation [133], predispose to liver fat accumulation in Indian men by altering lipid metabolism and IR [134]. The relationship with altered liver enzymes and liver damage was not assessed. This association was not replicated by a later study which found no association between these two ApoC3 polymorphisms and hepatic triglyceride content, IR or fasting triglycerides levels in a large multi-ethnic US cohort [135]. Neither was this validated in an Italian study of 585 obese subjects [136]. Furthermore, *APOC3* genotype was not associated with elevated liver enzymes or with the histological severity of liver damage in Italian and UK patients [137], suggesting that the initial observation may represent a type 1 error. 

### 6.2 Variants Involved in the Pathogenesis of IR

**Fig. (5):**
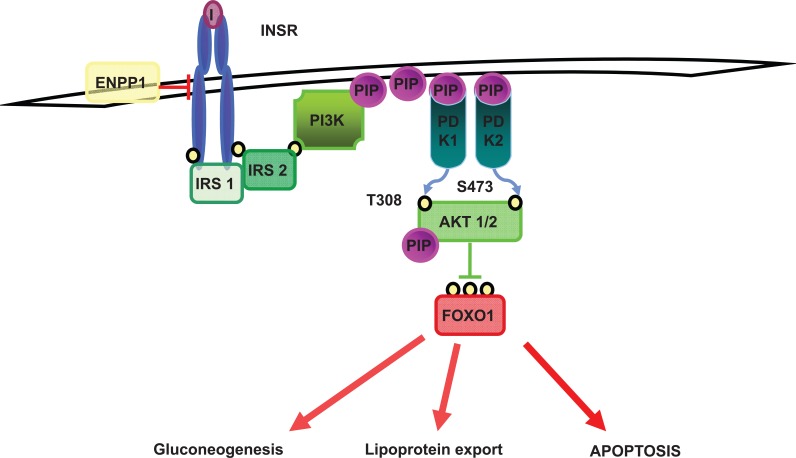
The insulin signalling pathway in hepatocytes. Insulin (I) binding to the extracellular subunits of insulin receptor (InsR) leads to activation of tyrosine kinase in the intracellular domain, adenosine triphosphate (ATP) binding and finally receptor autophosphorylation. ENPP1 is a membrane glycoprotein that interact with InsR inhibiting its kinase activity. InsR autophosphorylation is followed by phosphorylation of the insulin-receptor substrates (IRS), activation of phosphoinositide 3-kinase (PI3-kinase) and subsequent phosphorylation of Akt/PKB (protein kinase B), which are involved in mediating the metabolic effect of insulin. FOXO1 is a transcription factor that in the absence of insulin induces gluconeogenesis, lipoprotein export, and apoptosis. Insulin-mediated Akt phosphorylation of FOXO1 leads to its nuclear exclusion, ubiquitination, and subsequent proteasomal degradation.

IR is the key factor in NAFLD pathophysiology and is deeply entangled with the progression of liver disease [138], but the causal relationship between IR and fibrogenesis remains unclear. Functional common SNPs of genes included in the insulin signalling pathway influence IR and the susceptibility to type 2 diabetes. A schematic representation of the insulin signalling pathway in the liver is presented in (Fig. **5**). Plasma cell antigen-1, also known as ENPP1 is a membrane glycoprotein, which inhibits insulin signalling. The ENPP1 Lys121Gln gain-of-function polymorphism enhances the interaction between the ENPP1 glycoprotein and insulin receptor (INSR), contrasting INSR kinase activity, and is associated with an increased diabetes risk [139]. IRS-1 transduces INSR signalling to downstream kinases regulating glucose and lipid metabolism, cell survival, and proliferation. The loss-of-function Gly972Arg SNP decreases IRS-1 activity and inhibits INSR autophosphorylation and activity [140], increasing the risk of IR and diabetes [141, 142]. We recently demonstrated that the combination of ENPP1 121Gln and IRS-1 972Arg alleles was associated with decreased activation of the insulin signalling pathway in the liver and influenced fibrosis severity in a large multicentre series of NAFLD patients [9]. These data suggest that hepatic IR has a causal role in the progression of liver damage in NASH and therefore, that amelioration of IR may improve the long-term progression of the disease. However, additional independent studies are required to replicate this association and to establish the efficacy of insulin sensitizers in ameliorating the progression of liver fibrosis. 

Adiponectin, the major adipokine that has insulin-sensitizing, anti-inflammatory and anti-fibrotic effects [143], and whose decreased levels have been shown to correlate with hepatic fat accumulation [144-146], is another molecule of interest. Increased adipose tissue IR leading to reduced adiponectin levels has been described in patients with severe NASH and hyperglicemia compared to healthy controls independently of body mass [84, 85]. Several papers have reported a significant decrease in the serum levels of adiponectin in NASH patients [147, 148], and the overall evidence support the existence of an inverse relationship between adiponectin levels and the severity of NAFLD [149-151], suggesting a role for adiponectin dysregulation in the pathogenesis of NASH [149]. Nevertheless, the available evidence on the correlation between adiponectin genetic variants and the progression of NAFLD is still debated. Tokushige *et al.* reported that two SNPs (+45T>G of exon 2 and +276G>T of intron 2) were associated with the progression of liver fibrosis and insulin resistance in NAFLD Japanese patients [152]. Musso *et al.* added that the same adiponectin SNPs modulate the acute adiponectin response to dietary fat, and are associated with the presence of NAFLD in the Italian population. Moreover, +45 TT and +276 GT/TT carriers had significantly increased prevalence and severity in NAFLD than in other genotypes [153]. Wang and colleagues observed hypo-adiponectinemia and insulin resistance in Chinese NAFLD patients with metabolic syndrome. However, they concluded that the T45G and G276T SNPs were not important determinants of NAFLD, even if they might influence serum ALT, BMI, insulin resistance, lipid metabolism, and plasma adiponectin concentration [154]. Interestingly, it seems that genetic variation in the hepatocellular receptor of adiponectin (ADIPOR2) may also influence liver fat content in Northern European subjects [155].

### 6.3 Variants Influencing Redox Status and the Stress Response

Increased FFA flux to the liver on a background of IR plays a key role in the pathogenesis of NASH through hepatocellular oxidative stress, which leads to reactive oxygen species (ROS) production during FFA oxidation. 

The mitochondrial enzyme manganese-dependent superoxide dismutase (MnSOD) encoded by the nuclear SOD2 gene, plays an important role in protecting cells from superoxide radicals [156]. A common polymorphism in the SOD2 gene (C47T, rs4880) results in an amino acid substitution (Ala16Val) in the signal sequence targeting the enzyme to the mitochondrial matrix, where it exerts its function [157]. The Ala allele has been associated with more efficient protein import and with a higher enzyme activity [158, 159]. Therefore, the SOD2 rs4880 polymorphism has been investigated as a possible susceptibility factor in NASH and several other diseases related to oxidative stress including hereditary hemochromatosis [160]. In the largest available study in NAFLD, Al-Serri *et al*. examined a possible association between SOD2 genotype and susceptibility to NASH using two complementary approaches: a family study in which they analyzed trios consisting of children with fibrotic NAFLD and their two parents, and a classical case-control allelic association study in unrelated patients with NAFLD of varying severity. Using both the methodologies, a consistent association between the C47T SNP and fibrosis was demonstrated, providing persuasive genetic evidence that mitochondria-derived oxidative stress is important in the pathogenesis of advanced NAFLD [11]. Consistent with these findings, Namikawa *et al*. reported that Japanese patients with NASH had a higher incidence of the T/T SOD2 genotype [121].

Further evidence supporting a role for mitochondrial ROS comes from the evaluation of uncoupling protein 3 (UCP3), a mitochondrial transporter that uncouples the oxidative phosphorylation by increasing the proton leak of the inner mitochondrial membrane. Some studies have pointed to a role for UCP3 in the regulation of whole body energy homeostasis [161], diet induced obesity [162] and regulation of lipids substrates [163]. A SNP in the UCP3 promoter (-55C>T, rs1800849) has been associated with increased expression of UCP3 mRNA in the skeletal muscle of Pima Indians [164] as well as with body mass [165] and an atherogenic lipid profile in French Caucasians [166]. More relevant, in NAFLD patients, the rs1800849 UCP3 -55CT genotype was associated with insulin resistance, adiponectin levels, the presence of moderate-severe steatosis and NASH [167].

It has been suggested that excess hepatic iron deposition, a frequent feature observed in patients with NAFLD, may contribute to oxidative stress within the liver [22, 168]. The C282Y and H63D mutations of the hemochromatosis (HFE) gene represent a common cause of inherited iron overload in individuals of European ancestry [169]. The mechanism is related to decrease hepcidin release leading to increased iron absorption and parenchymal accumulation [170]. Although initial studies reported that mild iron overload associated with heterozygosity for C282Y HFE mutation may confer susceptibility to NAFLD and cause relative insulin deficiency [171], the available literature suggests that HFE mutations do not predispose to steatosis [172]. It is conceivable that HFE mutations could contribute to oxidative stress via hepatic iron loading among patients with NASH, and increase the susceptibility to fibrosis progression however this is not entirely supported. The relationship between HFE mutations and liver fibrosis is controversial too. Initial reports suggested that increased ferritin levels were markers of histological damage, but that *HFE* mutations did not contribute to hepatic fibrosis in many patients with NAFLD [173]. Later, Nelson *et al.* suggested that the presence of the C282Y mutation was a risk factor for development of advanced hepatic fibrosis among US Caucasian patients with NASH [174]. More recently, two large multi-centre studies conducted in Northern Italy and the US again reported that iron deposition was a risk factor for moderate/severe fibrosis in patients with NAFLD, but that *HFE* genotype determination was not clinically useful in these patients unless evidence of severe parenchymal iron accumulation is obtained [175, 176]. As other genetic factors more reliably predicted hepatic iron accumulation, e.g. the beta-thalassemia trait [10], it is likely that a wider panel of genetic variants influencing iron metabolism would be required to refine the risk of progressive disease. Independent of the genetic background, iron overload could be an appealing therapeutic target in some patients with NASH but this remains to be realised [168]. 

Finally, endoplasmic reticulum stress and the activation of the unfolded protein response has been implicated in NASH pathogenesis independently of oxidative stress [177]. Alpha-1-antitrypsin (AAT), is the principal serum protease inhibitor synthesized by the liver. Several variants of this gene have been described, the most common being the PiZ (Glu342Lys) and PiS (Glu264Val) alleles, whose prevalence is about 1% and 4% respectively in Northern Italy and show a decreasing gradient from North to South in Europe [178, 179], and potentially represent genetic modifiers of hepatocellular damage and inflammation. These amino acid substitutions lead to abnormal folding and spontaneous protein polymerization, determining endoplasmic reticulum stress and hepatocellular damage. Heterozygosity for the PiZ and to a lesser extent for the PiS alleles has been associated with cirrhosis and hepatocellular carcinoma (HCC) [180, 181]. In Italian patients with NAFLD, the presence of the PiS and PiZ alleles was associated with hyperferritinemia and non-parenchymal iron accumulation, likely in response to the activation of the unfolded protein response in the endoplasmic reticulum [182], but on the other hand were not associated with more severe liver disease [183]. However, whether AAT mutations predispose to hepatocellular carcinoma in patients with NAFLD remains to be evaluated.

### 6.4 Variants Influencing Inflammation

Obesity and NAFLD are associated with the increased production of cytokines by hepatocytes and Kupffer cells in response to bacterial products of intestinal origin [24], leading to hepatic and systemic IR, and contributing to the progression from steatosis to NASH [2].

Toll-like receptor 4 (TLR4) is a transmembrane receptor which signals through adaptor proteins in activating downstream effectors that include nuclear factor kB (NF-kB) [184], mitogen-activated protein kinase, and phosphatidylinositol 3-kinase (PI3K) [185] that control cell survival and apoptosis [186], and plays a critical role in mediating the activation of Kupffer cells in the response to LPS in NAFLD [23, 187]. In the liver TLR4 signalling contributes to hepatic inflammation and injury in NAFLD [23, 188, 189]. The T399I (1196C>T) SNP of TLR4 gene emerged as conferring protection from fibrosis progression [190] along with the highly co-segregated D229G (896A>G) polymorphism. The TLR4 T399I and D299G are two common, highly linked non-synonymous SNPs within the extracellular domain of TLR4 protein, which may affect the strength of interactions with either agonists and/or co-receptors [191]. Guo *et al.* demonstrated that TLR4 D229G and T399I SNPs that are associated with protection from hepatic fibrosis reduce TLR4-mediated inflammatory and fibrogenic signalling, and lower the apoptotic threshold of activated hepatic stellate cells (HSCs) [192], thus suggesting a critical role of TLR4 signalling in regulating HSCs activation. 

Evidence indicating that apoptosis is the major pathway of cell death during NASH make TNFα, a pro-inflammatory cytokine, a good candidate for a role in mediating liver injury given its ability to induce apoptosis in hepatocytes under conditions of oxidative stress and to induce IR. Two polymorphisms in the TNFα promoter region have been studied more extensively: one at position 308 (TNF2 allele) [193] and another at position 238 (TNFA allele) [194]. TNF2 allele is associated to increased constitutive and inducible expression of TNFα [195, 196]. Conflicting data have been reported on the TNFA allele [197], but most investigators believe that TNFA allele is associated to an increased release of this cytokine. Increasing evidence suggests that TNFα is involved in the pathogenesis and progression of liver disease of different aetiology [198-202]. TNFα SNPs have been reported to influence susceptibility to several hepatic diseases including alcoholic steatohepatitis [197] as TNFα appears to be involved in both the early stage of fatty liver disease and also the transition to steatohepatitis and more advanced stages of liver damage [203]. Conflicting data have been reported on the association of TNFα polymorphisms, serum insulin levels, IR index, per cent body fat, and type 2 diabetes mellitus [204-206]. The prevalence of the -238 TNFα polymorphism was reportedly higher in Italian patients with NAFLD than in controls, and TNFα polymorphisms were associated with IR, pancreatic β-cell function, and NASH [207]. Pastor *et al.* found that the TNFA allele is associated with a higher risk to develop liver cirrhosis in a Spanish alcoholic cohort [208]. In contrast, in a prospective cohort of Chinese patients with histology-proven NAFLD, TNFα polymorphisms were not associated with either presence of NAFLD or disease severity [209]. HCV infection is also associated with increased production of TNFα [210]. Our group reported that in patients with HCV chronic hepatitis TNFα genotype modulates the activity of the cytokine pathway, influences insulin sensitivity and the severity of HCV chronic hepatitis, but not liver steatosis [211]. On the contrary, Sanchez-Munoz *et al*. did not find any difference in insulin resistance, β-cell reserve, insulin and leptin levels between HCV patients with or without mutation at the promoter region of the TNFα gene [212]. All in all, results are not consistent across the populations evaluated, and therefore it is likely that the reported associations are explained by the extensive linkage disequilibrium and genetic variability within the HLA-C region including the TNFα gene, determining the associations of different TNFα alleles with other causal variants near the *TNFA* locus.

Interleukin 6 (IL-6) is another cytokine involved in both inflammation and IR [213]. However, whether specific IL-6 SNPs are associated with IR remains still disputed, given that conflicting results have been reported, probably due to population specific differences in the predisposition to IR and type 2 diabetes. The -174G/C promoter SNP has been reported to have either a protective or a promoting role for the development of type 2 diabetes [214, 215]. Several studies have shown that C allele is associated with IR, diabetes, and metabolic syndrome [215-217]. Carulli *et al.* found that the IL-6 -174C variant was significantly more prevalent in NAFLD than in healthy subjects, was associated with increased fasting insulin and HOMA-IR, and was an independent predictor of NAFLD and NASH [218]. However, this study considered a very limited number of NAFLD patients of whom only half had had liver biopsy [218].

### 6.5 *IL28B* Genotype, Steatosis, and NASH

Genome-wide association studies have recently identified genetic variations near the *IL28B* gene, encoding for interferon (IFN)-λ3, as a strong predictor of spontaneous and treatment-induced clearance of hepatitis C viral infection [219-224]. Protective variants at the rs12979860 and rs8099917 loci have consistently been associated with faster decline of viral load and an approximately two-fold higher sustained virologic response rate during standard of care treatment, in particular in patients affected by the difficult to cure HCV genotype 1 and 4 [225, 226]. The mechanism by which these genetic variants influence the outcome of HCV infection, i.e. whether they influence IFN-λ3 expression by affecting gene transcription or are linked a coding variant (Lys70Arg) of the IFN-λ3 protein, is still debated [220, 224], but it seems to result in a different pattern of activation of the innate immune system against HCV infection, as determined by the different basal and IFN-α induced expression of interferon stimulated genes and inflammatory activity [227, 228], possibly influencing viral evolution under the selective pressure of the immune system [229]. 

Steatosis is frequently observed in patients with chronic hepatitis C (CHC), particularly those with genotype 3 infection, and is associated with fibrosis progression and treatment failure [230, 231]. Tillmann *et al.* recently reported a negative association between the *interleukin 28B (IL28B)* rs12979860 CC genotype, predicting sustained virological response [219], and steatosis in genotype-1 CHC [232]. Therefore, through the effect of inflammatory cytokines on lipid metabolism or by favouring a better control of HCV replication [211], *IL28B* favourable variants protect from the development of steatosis [232], and possibly from the steatosis-associated fibrosis progression and increased risk of HCC [230, 233]. In addition, the negative association between the CC genotype and steatosis may partly explain the association of steatosis with resistance to peginterferon plus ribavirin therapy in genotype 1 CHC patients. However, the effect of *IL28B* variants likely on fibrosis progression rate in patients with ongoing HCV was still controversial [231, 234, 235]. 

As discussed in the previous paragraphs, the *PNPLA3 *rs738409 polymorphism is a strong determinant of hepatic fat accumulation and steatohepatitis [8, 52], but also influences steatosis and fibrosis progression in CHC [64, 233, 236]. A previous study [236], also reported an association between *IL28B* rs12980275 genotype and steatosis in CHC non genotype 3 patients, but the rs12979860 *IL28B* polymorphism was not tested and the interaction with *PNPLA3* genotype was not analyzed in details. The negative association of rs12979860 CC with histologically-determined steatosis was recently confirmed in 567 naïve, consecutive, non-genotype 3 patients from referral centres in Milan and Vienna, without excessive alcohol intake [237]. The association between *IL28B* genotype and steatosis was independent of acquired risk factors, and of the *PNPLA3* GG genotype. Interestingly however, the rs12979860 CC genotype protected form steatosis in patients positive, but not in those negative for the *PNPLA3* G variant at risk, suggesting that an interaction occurs between *IL28B* and *PNPLA3* genotypes in the pathogenesis of steatosis in CHC non genotype-3 patients. In the same cohort of patients, a significant interaction between the rs12979860 *IL28B* CC and PNPLA3 genotype on liver damage was also observed, as the *IL28B* CC genotype was associated with advanced fibrosis only in patients negative for the *PNPLA3* GG genotype independently of age, BMI, and ALT levels [65]. These data indicate that stratification for *PNPLA3* GG genotype unmasked an association between IL28B CC genotype and more severe liver fibrosis, which may be related to increased hepatic inflammation associated with the favourable rs12879860 allele.

Interestingly enough, Petta *et al*. have recently reported that in 160 consecutive patients with biopsy-proven NAFLD, the *IL28B* rs12979860 CC genotype was not associated with protection from steatosis in the absence of viral infection, but it was associated with about a four-fold increased risk of moderate-severe lobular inflammation independently of age, gender, triglycerides, hyperuricemia, and steatosis grade, and was significantly associated with severe fibrosis (stage 3-4) at univariate analysis [238]. Intriguingly, the risk of more severe inflammation conferred by the “at risk” CC variant was particularly evident in subjects carrying also the PNPLA3 G allele. Provided that the association between *IL28B* genotype and hepatic inflammation complicating NAFLD is confirmed in larger series that are urgently awaited, data would suggest that the *IL28B *CC genotype represent a host factor influencing hepatic inflammation in different liver diseases, and the incorporation together with PNPLA3 in non-invasive scores could be useful to refine the risk of NASH.

### 6.6 Variants Involved in HSCs Activation and Fibrogenesis

Activated HSCs are the major source of extracellular matrix (ECM) deposition during fibrogenesis [239]. HSCs also release fibrogenic cytokines with autocrine and paracrine effects, including TGF-β1, and over-express tissue inhibitors of metalloproteinase, which promote ECM accumulation by inhibiting matrix degradation.

Kruppel-like factor 6 (KLF6) belongs to the Kruppel-like family of transcription factors that play diverse roles in differentiation, cell growth, apoptosis and angiogenesis [240]. KLF6 was identified as an early gene expressed in activated hepatic stellate cells (HSCs) after liver injury [241, 242], raising the possibility that it may be involved in the process of liver fibrogenesis. Indeed, KLF6 transactivates several genes critical for the development of liver fibrosis, including collagen 1, TGF-β1 and types I and II TGF- β receptors in HSCs [242], [243]. A functional SNP, the IVS1-27G>A SNP (rs3750861) located within the first intron, has been identified in the KLF6 gene [244]. Miele *et al.* showed that the presence of the KLF6 IVS1-27G>A SNP, which was demonstrated to reduce fibrogenesis in HSCs, was associated with less fibrosis in a UK cohort with biopsy-proven NAFLD patients. This trend was confirmed in an independent group of Italian patients. Moreover, analysis of the combined UK and Italian groups identified the presence of wild-type KLF6 as a predictor of moderate/advanced fibrosis independently of all other risk factors of progressive disease, suggesting that the wild-type KLF6 genotype is a significant susceptibility factor for fibrotic NAFLD, whereas KLF6 IVS1-27G>A protects against the development of fibrosis [12]. The effect of KLF6 genotype on NASH might not be limited to modulation of fibrogenesis, as it also influenced fasting glucose levels. Bechmann *et al.* observed that KLF6 IVS1-27G wild-type allele was associated with stepwise increase in fasting plasma glucose and insulin and reduced hepatic insulin sensitivity [245], and the effect was at least partially mediated by reduced expression of glucokinase, raising the possibility that the effect of this variant on the progression of liver damage in NASH might entail regulation of glucose and lipid metabolism.

The growth factor TGF-β1 also plays a dominant role in mediating hepatic fibrosis by contributing to the activation of HSCs [246, 247]. Several polymorphic sites have been described within the TGF- β1 gene. One non-synonymous SNP at codon 25 (+915) C/G, encoding an Arg25Pro substitution, modulates TGF- β1 production *in vitro* and occurs within the peptide signal sequence that is cleaved from the active TGF-β1 protein. Individuals with the Arg/Arg homozygous genotype produce substantially more TGF-β1 protein than individuals with the Arg/Pro genotype. In patients with chronic hepatitis C, those with the high TGF-β1-producing (Arg/Arg) at codon 25 were more likely to have increased hepatic fibrosis compared to subjects with Arg/Pro or Pro/Pro genotypes [248]. In addition, the pro-fibrotic Arg/Arg genotype was more frequent in patients with hypertension versus normotensive controls [249]. In cardiac and renal fibrosis, TGF-β1 production is triggered by angiotensin II (AII), the principal effector molecule of the renin-angiotensin system (RAS) [250, 251]. Recent findings indicate that AII may augment the accumulation of extracellular matrix [252]. An AT polymorphism in the promoter region of the gene (AT-6 G>A), which affects the basal transcription rate of the gene leading to increase AII production, was also associated with progressive hepatic fibrosis in Australian patients with chronic hepatitis C [248]. Since IR and systemic hypertension are predictors of advanced fibrosis in obese patients with NAFLD, Dixon *et al.* hypothesized that high AT and TGF-β1 producing genotypes increase the risk of liver fibrosis in obese subjects with NAFLD. In 105 severely obese subjects with NAFLD, they showed that individuals who inherited a combination of two pro-fibrotic genotypes have a significantly increased risk of advanced hepatic fibrosis [253].

### 6.7 Variants Influencing Telomerase Activity

Telomere diseases, exemplified by dykeratosis congenita, which is caused by mutations in DKC and other genes involved in telomeres maintenance, are characterized by premature senescence of the staminal compartment, tissue fibrosis due to the loss of regenerative capacity of tissues, and increased cancer risk due to chromosomal instability consequent to the faulty protection of chromosome ends, i.e. the telomeres, during mitosis [254]. Clinical features include hematological alterations ranging from macrocytosis to bone marrow failure, mucocutaneous alterations, pulmonary fibrosis, diabetes, and cirrhosis. Loss-of-function mutations in the telomerase gene (TERT) are also responsible in combination with environmental factors of a significant proportion of cases of familial idiopathic pulmonary fibrosis [255, 256].

Most importantly however, mutations in TERT and in TERC, encoding for the RNA primer of TERT, have been associated with a spectrum of familial hepatic liver disease often associated with histological steatosis similar to NAFLD and hepatic iron overload [257]. Furthermore, they have recently been demonstrated to represent a frequent risk factor for cirrhosis, being observed in 3-8% of unselected patients, in other liver diseases, including chronic viral hepatitis, alcoholic liver disease and also NAFLD [258, 259]. TERT over-expression is a frequent mechanism in HCC related to chronic hepatitis [260] because it favours the replicative potential of the stem cell compartment. HCC cases have already been reported in a few patients with TERT mutations [254, 259], suggesting that an alternative carcinogenic pathway likely involving chromosomal instability ensues, that is associated with aggressive biological features. Furthermore, rapid progression of liver cirrhosis, aggressive recurrence of HCC, and poor outcome after liver transplantation have been reported in patients with TERT mutations [259], suggesting that further studies are needed to define the optimal management of liver failure and HCC occurring in patients with TERT mutations [258, 259]. 

These findings suggest the opportunity to re-evaluate the most currently accepted models explaining the progression of liver damage during steatohepatitis and liver diseases, which are based only on the activation of fibrogenesis without taking into account the exhaustion of the parenchymal regenerative compartment, and establish genes involved in telomere maintenance as attractive targets for future genetic as well as therapeutic research. They also provide the first demonstration that relatively rare genetic variants with high penetrance, which by definition could not be detected by GWAS, are associated with the progression of common liver diseases including NAFLD. Given the recent technological developments allowing high-throughput sequencing at affordable costs in the near future, it is likely that the evaluation of the role of these variants in the progression of NASH to cirrhosis and hepatocellular carcinoma, and in the pathogenesis of cryptogenic cirrhosis will became an active area of research.

## 7 SPECIFIC ISSUES IN THE PAEDIATRIC POPULATION

Pediatric NAFLD has also become a common chronic liver disease in children and adolescents in industrialized countries following the growing prevalence of childhood obesity [261-263]. NAFLD affects 3-10% of subjects during the developmental age, and this figure increases up to approximately 80% among obese [263-266]. A large survey found elevated ALT in 8 % of U.S. adolescents [267], whereas in the two largest samples of biopsy-proven NAFLD, NASH was diagnosed in 64-84% of NAFLD children in Italy and California [268, 269]. Conditions predisposing to pediatric NAFLD are generally hyper-alimentation associated with inadequate physical activity leading to a progressive increase of body mass and visceral adiposity. Higher intake of calories than needed for growth may cause overweight and obesity in children. This is becoming more and more diffuse with the daily consumption of fast foods and soft drinks, associated with inactive leisure activities, such as watching television or playing video games. However, familial, epidemiological, and twin studies suggest that also inherited factors may play a pivotal role in determining the susceptibility to develop pediatric NASH [6, 7, 27, 34, 270]. 

As NAFLD has a major genetic component [6], due to the lower number of confounding factors, such as the duration of disease, presence of obesity, lifestyle habits, comorbidities, and drugs, and the likely more important role played by genetic factors in early-onset disease in the presence of environmental triggers such as obesity, this is especially true for children [54].

Indeed, the association between the I148M variant of *PNPLA3*, the major risk factor for NASH identified so far, and both liver enzymes and steatosis was soon confirmed in obese children of different ethnicity [55, 56, 271, 272], and in one family study in Italian trios [52], indicating that it exerts its effect early in life. Importantly, the magnitude of the association between the I148M *PNPLA3* variant and liver enzymes was shown to be related to the size of abdominal fat [60], and to high dietary carbohydrate and sugar consumption specifically during the developmental age [62]. Furthermore, *PNPLA3 *genotype influenced the histological severity of NASH alterations and fibrosis in obese pediatric patients who underwent biopsy because of persistently altered liver enzymes [54]. Interestingly, the association with fibrosis was stronger than in adults [50], in that, after adjustment for other risk factors such as age, waist circumference, hyperglycemia, and ALT levels, each 148M allele increased the risk of fibrosis by almost two-fold [54].

A more recent GWAS conducted in a larger population was able to identify a wider set of genetic variants influencing steatosis besides I148M of *PNPLA3* [273], of whom the rs2854116 SNP of *Glucokinase regulator* (*GCKR*), involved in the regulation of the uptake of monosaccharides and lipogenesis was confirmed to predispose to fatty liver and dyslipidemia in obese children and adolescents independently of *PNPLA3* [75], although the effect on histological progression of liver disease is still unknown, especially in view of the ameliorating effect on insulin resistance.

Additional variants in genes implicated in NASH pathogenesis have been shown to influence liver damage and fibrosis progression in candidate gene case-control studies using pediatric patients. These include genetic variants regulating insulin receptor activity, namely the *ENPP1* Lys121Gln and the *IRS-1* Gly972Arg functional SNPs [9], the *SOD2* C47T rs4880 SNP regulating SOD2 mitochondrial import and anti-oxidant activity [274], and the *KLF6* IVS1-27G>A SNP regulating alternative splicing isoforms of the transcription factor KLF6 involved in the regulation of the regulation of metabolism in hepatocytes and fibrogenesis in hepatic stellate cells [275]. In contrast, variants in the *APOC3* regulating VLDL metabolism were not confirmed to influence the susceptibility to steatosis and NASH [276].

Finally, there is growing awareness that the expression of some genetic variants may be age-dependent, i.e. that the phenotype might be more (or less) marked or involve different traits during the developmental age. For example, the common variant (rs13412852) influencing the expression of *Lipin-1* (*LPIN1*) [277], was associated with lipid levels, NASH severity, and hepatic fibrosis in children with NAFLD, whereas it influenced body mass, but not the severity of liver histology, in adults with NAFLD of the same ethnicity [278, 279].

To summarize, genetics has a key role in determining who among the large fraction of the pediatric population with metabolic risk factors will develop progressive liver disease [6]. The I148M variant of *PNPLA3* is likely the major genetic determinant of increased hepatic fat content by interacting with body fat and dietary factors [8, 54, 60, 62], but it also influences the susceptibility to NASH and fibrosis, [278]. Additional studies are required to validate these findings at population level and in prospective studies, to evaluate whether *PNPLA3* influences the response to therapy (see below), and to define the possible relevance of I148M genotype for the clinical management of patients, and in particular to develop new non-invasive scores that may avoid to perform liver biopsy that is especially problematic in young children. Evaluation of the interaction of *PNPLA3* with other genetic variants influencing steatosis and NASH, including *GCKR [75]*, *ENPP1* and *IRS-1 [9]*, *SOD2 [274]*, *KLF6 [275]*, *LPIN1 [278]*, and possibly other SNPs will be instrumental to achieve these goals.

## 8 INFLUENCE OF GENETICS ON TREATMENT OUTCOME

As the previous sections have highlighted, the genetic basis of susceptibility to NAFLD and progressive NASH is beginning to be elucidated, but very little is known about the effect of genetics on the response to treatment. Much of the uncertainty is obviously related to the lack of effective pharmacological treatment specific for NASH. However, lifestyle changes and in particular sustained weight loss has unequivocally demonstrated to improve histological features of NASH in the majority of patients [280, 281], suggesting that in most cases genetic risk factors are not able to cause NASH in the absence of environmental triggers. A recent systematic review and meta-analysis has shown that exercise interventions *per se* reduce liver fat despite minimal or no weight loss confirming a role for exercise as a therapeutic target in NAFLD [282].

The minor G allele of PNPLA3 has been suggested to impair triglyceride hydrolysis in *in vitro* studies and several studies have shown that the GG carriers have an increase risk of NASH, for which weight loss is considered the perhaps best treatment [283]. In a recent study Sevastianova *et al.* evaluated whether weight loss is able to decrease liver fat in homozygous carriers of the G allele (PNPLA3-148MM). They found that 148II and 148MM patients lost similar amounts of body weight in response to a 6-day hypocaloric, low carbohydrate diet. However, liver fat content decreased significantly more in the 148MM group than in the 148II one, although this was in part because of the higher baseline levels. These data suggest that weight is an effective means for reducing liver fat content in subjects with PNPLA3-148MM [284], highlighting that NAFLD is a complex trait exhibiting a strong interaction between genetic and acquired risk factors for NASH, and most importantly confirming that behavioural changes can counteract the effects of the strongest known inherited risk factor for progressive NASH [50]. Pending more definitive studies, these data could provide a rationale to support use of low carbohydrate diet in subjects with NASH that poses the 148MM PNPLA3 genotype. Unfortunately, there are no published data addressing the interaction between other genetic variants and weight loss. We do not know whether the effect of antioxidant therapies such as vitamin E, which may provide some benefit in a sub-set of patients [285], might be influenced by polymorphisms modulating oxidative stress response [11]. Although SNPs in the PPARγ gene influence IR, it is not known whether the effect of insulin sensitizing drugs such as glitazones that target this transcription factor and reduce steatosis in some patients with NASH [285] are influenced by PPARγ genotype.

Limited data are available on possible role of genetic factors in influencing the effect of iron depletion therapy. Indeed, hyperferritinemia reflecting increased body iron stores is frequently observed in NAFLD due to the association with steatosis and IR, and associated with faster progression of organ damage [168]. Iron depletion by phlebotomy has been reported to decrease both IR and liver enzymes in NAFLD patients more than lifestyle changes alone [286]. A retrospective study has also shown that iron depletion produced a significantly greater improvement in insulin sensitivity than nutritional counselling alone and that iron depletion was effective in reducing HOMA-R in patients with high ferritin concentrations and in carriers of *HFE *genetic mutations causing hereditary hemochromatosis [287], suggesting that *HFE *genotyping might be used to select subjects who might benefit most from this approach. However, the study was not designed to test this hypothesis and the outcome was not evaluated histologically, this therefore remains speculative.

## FUTURE DIRECTIONS

Despite the recent progresses, several key issues remains to be addressed in the next years, including, but not limited to:

1. The mechanism linking the I148M PNPLA3 variant with progressive liver disease, its role in other liver diseases, and the potential clinical utility of its determination for best tailoring the clinical management of the patients with NASH.
2. The validation of the association of other genetic variants associated with liver fat content in GWAS studies (e.g. GCKR) with the progression of liver diseases associated with steatosis.
3. The evaluation of risk factors associated with advanced disease in patients at risk undergoing liver biopsy by a GWAS approach.
4. The evaluation of the role of copy number variants and relatively rare gene variants associated with a high effect on the risk of progressive NASH.
5. The evaluation of the interaction between genetic and acquired risk factors in the pathogenesis of NASH.
6. The development and assessment of the utility diagnostic and prognostic scores incorporating multiple genetic risk factors associated with clinically relevant end-points.
7. The evaluation of the effect of genetic factors on the response to therapy, including specific diets and physical activity programs and drugs.

## 10 CONCLUSION

Genes play a key role in the development and progression of NAFLD by interacting with environmental factors. To date, PNPLA3 polymorphisms are the best validated susceptibility modifiers for steatosis and progressive hepatic injury. However, several other genetic variants that contribute to steatosis and/or steatohepatitis have been identified through GWAS studies, and risk factors of progressive NASH have been validated in other large multi-centre studies (Tables **1**-**3**). It is now important to explore the molecular mechanisms underlying these associations between gene variants and progressive liver disease, and to evaluate their impact on the response to available therapies. It is hoped that this knowledge will offer further insights into pathogenesis, suggest novel therapeutic targets, and help guide physicians towards individualised therapy that improves clinical outcome. 

## Figures and Tables

**Table 1.** Genetic variants influencing NAFLD susceptibility identified by genomewide scans (GWAS) [8, 77, 273].

Gene	SNP	Effect on steatosis	Effect on NASH/fibrosis/inflammation
PNPLA3, patatine-like phospholipase domain containing 3 [48]	rs738409 rs6006460 [8]	↑ ↓	↑­
FDFT1, farnesyl diphosphate farnesyl transferase 1	rs2645424		↑­
COL13A1, collagen, type XIII, alpha1	rs1227756		↑­
EFCAB4B, EF-hand calcium binding domain 4B	rs887304		↑­
NCAN, neurocan	rs2228603	↑­	
LYPLAL1, lysophospholipase-like 1	rs12137855	↑­	
GCKR, glucokinase regulatory protein	rs780094	↑­	
PPP1R3B, protein phosphatase 1, regulatory subunit 3b	rs4240624	­↑	

**Table 2.** Genetic risk factors for NAFLD evaluated in case-control studies.

Gene	SNP

*Genetic variants involved in the modulation of steatosis*	
PEMT, phosphatidylethanolamine N-methyltransferase [113]	rs7946
PPARα, peroxisome proliferative activated receptor alpha [81]	rs1800234
PPARγ, peroxisome proliferative activated receptor gamma [83, 87]	rs1805192
APOE, apolipoprotein E	N/A
APOC3, apolipoprotein C-III [288]	rs2854116
	rs2854117

*Genetic variants involved in glucose metabolism*	
ADIPOQ, adiponectin [153]	rs2241766
	rs1501299

*Genetic variants influencing redox status and stress response*	
SOD2, superoxide dismutase 2, mitochondrial [274]	rs4880
UCP3, uncoupling protein 3, mitochondrial [167]	rs1800849

*Genes influencing inflammation*	
TNFα, tumor necrosis factor alpha [207]	rs361525
IL-6, interleukin [218]	rs1800795
IL28B [230, 232, 233, 238, 289]	rs12979860

N/A: not available

**Table 3.** Genetic risk factors for progressive liver disease in NAFLD evaluated in case-control studies.

Gene	SNP

*Genetic variants involved in modulation of steatosis*	
MTTP, microsomal triglyceride transfer protein [121, 122, 124, 125]	rs1800591
PEMT, phosphatidylethanolamine N-methyltransferase [113, 114]	rs7946
PPARγ, peroxisome proliferative activated receptor gamma [88]	rs1805192
APOE, apolipoprotein E [129]	N/A
LPIN1, lipin 1 [94]	rs13412852

*Genetic variants involved in glucose metabolism*	
ENPP1, ectonucleotide pyrophosphatase/phosphodiesterase1 or PC-1 [9]	rs1044498
IRS1, insulin receptor substrate 1 [9]	rs1801278
ADIPOQ, adiponectin [152]	rs2241766
	rs1501299

*Genetic variants influencing redox status and stress response*	
SOD2, superoxide dismutase 2, mitochondrial [11, 160]	rs4880
UCP3, uncoupling protein 3, mitochondrial [167]	rs1800849
HFE, hemochromatosis [175, 290]	rs1800562
	rs1799945

*Genes influencing inflammation*	
TNFα, tumor necrosis factor alpha [207]	rs361525
IL-6, interleukin [218]	rs1800795
TLR4, toll-like receptor 4 [192]	rs4986791
IL28B [65, 230, 233, 237, 238]	rs12979860

*Genetic variants involved in HSCs activation and fibrogenesis*	
KLF6, kruppel-like factor 6 [275]	rs3750861
TGF-β1, transforming growth factor beta [248, 253]	rs1800471

N/A: not available
